# Abelmoschus esculentus subfractions attenuate beta amyloid-induced neuron apoptosis by regulating DPP-4 with improving insulin resistance signals

**DOI:** 10.1371/journal.pone.0217400

**Published:** 2019-06-25

**Authors:** Chien-Ning Huang, Chau-Jong Wang, Chih-Li Lin, An-Ting Yen, Hsin-Hua Li, Chiung-Huei Peng

**Affiliations:** 1 Department of Internal Medicine, Chung-Shan Medical University Hospital, Taichung, Taiwan; 2 Institute of Medicine, Chung-Shan Medical University, Taichung, Taiwan; 3 Institute of Biochemistry, Microbiology and Immunology, Chung-Shan Medical University, Taichung, Taiwan; 4 Division of Basic Medical Science, Hungkuang University, Taichung City, Taiwan; University of Pécs Medical School, HUNGARY

## Abstract

The association of Alzheimer disease (AD) and Diabetes (DM) is less clear. Accumulation of beta amyloid (Aβ) and presence of hyperphosphorylated tau (p-tau) are hallmarks of AD, spreading in the region where insulin receptors are also found. Aβ exerts neuron toxicity, and could disturb insulin signaling of phosphatidylinositol 3-kinase (PI3K), glycogen synthase kinase (GSK)-3β and AMP-activated protein kinase (AMPK), but increase IRS-1-Ser307 phosphorylation which is viewed as insulin resistance marker. Previously we reported dipeptidyl peptidase-4 (DPP-4) mediate insulin resistance signals, and Abelmoschus esculentus (AE) subfractions F1 (rich in quercetin glucosides and triterpene ester) and F2 (containing large amount of polysaccharides) attenuate DPP-4-mediated apoptosis. In the present study, we aim to investigate if Aβ induce neuron death by regulating DPP-4 and insulin resistance signals, and the putative effect of F1 and F2. By MTT, microscopy, and Western blotting, we demonstrate treatment of appropriate doses of AE subfractions prevent Aβ-induced neuron apoptosis. F1 attenuate Aβ-induced caspase 3 expression especially at 25 μg/mL, while F2 attenuate caspase 3 activation even at the low dose of 1 μg/mL. Both AE subfractions decrease Aβ-enhanced DPP-4, but increase Aβ-reduced p-AMPK and p-PI3K. The activity analysis reveals that F2 is more valid than F1 to reduce DPP-4 activity. The inhibition of DPP-4 demonstrates it plays the pivotal role in Aβ-induced neuron apoptosis. Moreover, although both F1 and F2 are effective to inhibit p-IRS-1-Ser307, F2 takes advantage to reduce p-Tau while F1 is superior to enhance p-GSK-3β. This implies AE subfractions act on different targets, and could be developed respectively. In conclusion, we demonstrate AE is potential to prevent Aβ-induced neuron damage by regulating DPP-4 and the insulin resistance cascades. AE could be an adjuvant to protect neuron degenerative disease related to Aβ and insulin resistance.

## Introduction

The worldwide increasing prevalence of diabetes (DM), characterized by insulin secretion defect, insulin resistance or both, burdens the public health. As well, the incidence of Alzheimer disease (AD) is lifting at alarming rate in many developed countries [1]. Interestingly, the epidemiological studies showed that diabetic individuals have significantly higher risk in the development of AD [2, 3]. Although the cerebrovascular damage caused by DM could influence cognition, the clinical observation suggested that the association of AD and DM is independent of vascular factors, while the underlying mechanisms for this association were still not clear [4].

One of the neuropathological hallmarks of AD is the accumulation of beta amyloid (Aβ) peptide, a 38- to 43-amino acid peptide which is produced by sequential cleavage of amyloid precursor protein (APP) thus form the major component of senile plaques. An imbalance between production and clearance of Aβ42 is a very early and often initiating factor in AD. Another hallmark of AD is the presence of intracellular neurofibrillary tangle, which is composed of hyperphosphorylated tau (p-tau), a group of protein assembly and stabilize the microtubules. Aβ and p-tau spread in a progressive manner, followed by the substantial neuron apoptosis with characteristic “regional specificity” [5]. This pathological process may result in memory impairment, the main symptom of AD.

In fact, besides peripheral organs, insulin receptors are also found in central nervous system especially the regions of hippocampus and cerebral cortex, which play a pivotal role in learning and memory [6]. The insulin of brain may come from uptake of peripheral circulation through the blood brain barrier [6], or be produced endogenously [1]. Insulin action involves a series of signaling cascades initiated by binding to its receptor [7]. By eliciting tyrosine phosphorylation of insulin receptor substrates (IRS), the insulin action leads to activation of phosphatidylinositol 3-kinase (PI3K) and Akt [7], which phosphorylates important substrates including glycogen synthase kinase (GSK)-3β [8]. The AMP-activated protein kinase (AMPK) also be critically associated with the downstream cascades [9]. However, an increase in phosphorylation of IRS-1-Ser307 hinders the response signals and glucose utilization, thus viewed as the insulin resistance marker [7]. Recently, dipeptidyl peptidase-4 (DPP-4) inhibitors have emerged as useful tools for treating type 2 diabetes. The mechanism relies on inhibiting the degradation of type 1 glucagon-like peptide (GLP-1), an incretin binds to the receptor GLP-1R, thus stimulates glucose-dependent insulin secretion from β cells [10]. Our previous study have shown that DPP-4 mediate the insulin resistance signals and the downstream cascades [11].

It was shown that Aβ, as a direct competitive inhibitor, reduced the binding of insulin and activation of IRS [12]. In the cultured cells, Aβ induced insulin resistance through the downregulation of IRS [13]. These evidences suggested that Aβ could disturb the insulin signaling pathway by regulating signaling molecules mentioned above. For exploring the strategy to prevent the incidence of AD, whether Aβ mediates or burdens the neuron death via insulin resistance cascades will be never too much investigated.

The fruit of Abelmoschus esculentus (AE; commonly known as okra) is consumed as vegetables [14], and used in folklore medicine particularly for its anti-diabetic effect [15]. However, the substantial mucilage of AE makes it difficult to test the active components. We successfully isolated several subfractions from AE using a series of successive extraction steps, and found that the subfractions F1 (rich in quercetin glucosides and pentacyclic triterpene ester) and F2 (containing large amounts of carbohydrates and polysaccharides) were especially effective in suppressing DPP-4 signaling [16]. In our recent report, F1 and F2 are useful to prevent the apoptosis of β cells via regulating AMPK/mTOR, PI3K, and the activation of caspase 3 [17].

Hence in the present study, we aim to investigate if Aβ-induced neuron death by regulating GLP-1/DPP-4 and the insulin resistance-signals, and the putative action of F1 and F2.

## Experimental

### Preparation of AE subfractions and chemical analysis

AE was purchased from Chuchi, Chiayi. The subfractions of AE F1 and F2 were prepared according to a succession of procedures [16] [S1 File]. F1, the alcohol-extracted fraction of AE, was previously analyzed with LC-MS/MS. At least 10 compounds were composed of F1, including quercetin glucosides (4.901 mg/g DW) and pentacyclic triterpene ester (4.301 mg/g DW) [16]. The F2 portion of AE contains a large amount of polysaccharides. With a GPC analysis, the mean molecular weight of F2 was estimated to be 671 kDa. The monosaccharide analysis and uronic determination revealed that F2 is rich in uronic acid (23.14%), galactose (18.92%), glucose (18.26%) and myo-inositol (14.21%); rhamnose, glucosamine, and fucose were also found to be quite abundant [16].

### Preparation of Aβ42

Aβ42 was custom-synthesized by LifeTein (Somerset, NJ, USA) according to the sequence “DAEFRHDSGYEVHHQKLVFAAEDVGSNKGAIIGLMVGGVVIA”. The molecular weight is 4438. Aβ42 (purinity 95.11%) was prepared by dissolving phosphate buffer saline solutions in the following experiments.

### Cell culture

The cell line SK-N-MC has been selected to demonstrate the role of DPP-4 in Aβ‐induced cytotoxicity in our previous report [18]. SK-N-MC was from American Type Culture Collection (ATCC). The cells were cultured in Minimum Essential Medium (MEM) with 2mM L-glutamine, penicillin-streptomycin and 10% fetal bovine serum (FBS), and grown in a humidified incubator at 37°C in an atmosphere of 5% CO2 and 95%. The medium was changed every 2–3 days. When the cells were approximately 80% confluent, the subcultures were undergone. Phase contrast microscope with photo system was used for image observation.

### MTT

Cells were seeded at a density of 2 × 10^5^ cells/mL in a 24-well plate. After attached, cells were incubated with Aβ42 and each AE fraction at various concentrations for 24 h. The medium was then changed and cells were incubated with 3-(4, 5-dimethylthiazol-2-yl)-2,5diphenyltetrazolium bromide (MTT, 0.5 mg/mL) for 2 h. The viable cell was directly proportional to the production of formazan. Following the dissolution in isopropanol (1mL/well), being centrifuged (at 12000rpm), Each sample was added 200 μL per well into a 96-well plate, then red with spectrophotometer at 563 nm (Hitachi, U-3210).

### Western blot

Cells were harvested into lysis buffer containing 50 mM TrisHCl (pH 6.8), 10% glycerol, 2% SDS, and 5% mercaptoethanol, and then lysed by sonication. Cell lysate was centrifuged at 9300g for 20 min at 4°C, and the supernatant was collected as the protein sample. After quantification, equal amounts of protein samples (50 μg) were subjected to 10% SDS-polyacrylamide gel electrophoresis and transferred to nitrocellulose membranes (Millipore, Bedford, MA, USA). Membranes were blocked with 5% nonfat milk powder with 0.1% Tween-20 in TBS, and then incubated with the primary antibody at 4°C overnight against the following targets: cleaved caspase 3 (1:500), procaspase 3 (1:200), GLP-1R (1:1000), DPP4 (1:5000), pPI3K (1:200), PI3K (1:200), pAMPK (1:1000), AMPK (1:200), p-IRS-1-Ser307 (1:1000), p-Tau (1:1000) and p-GSK3 (1:1000). Antibodies of cleaved caspase 3 and GLP-1R were from Abcam. DPP4 was from Novus. Pro-cas3, PI3K and AMPK, and p-GSK3 were from Santa Cruz. p-PI3K and p-AMPK were from Bioss and Cell Signaling, respectively. p-IRS-1-Ser307 and p-Tau were from Merk. After this, membranes were washed three times with 0.1% Tween-20 in TBS and incubated with the secondary antibody (1:5000) conjugated to horseradish peroxidase (GE Healthcare, Little Chalfont, Buckinghamshire, UK). Band detection was thereafter revealed by enhanced chemiluminescence using ECL western blotting detection reagents and exposed in FUJFILM Las-3000 (Tokyo, Japan). Protein quantity was determined by densitometry using FUJFILM-Multi Gauge V2.2 software.

### DPP-4 activity

Cells were seeded on 6-cm dish at the density of 1 × 10^6^. After treatment under various conditions, cells of each well were lysed with 100 μL of NP-40 lysis buffer (containing 10 mM HEPES (pH 7.5), 142.5 mM KCl, 5 mM MgCl2, 1 mM EGTA, and 0.2% NP-40) and centrifuged at 9300*g* for 20 min at 4°C. The supernatants were collected, and the protein concentrations were determined with the Bradford assay. DPP4 activity was measured using the DPP4/CD26 assay kit for biological samples (Enzo Life Sciences). Briefly, H-Gly–Pro-pNA, a chromogenic substrate of DPP-4, was hydrolysed into dipeptide Gly–Pro and 4-nitroaniline, whose rate of appearance was measured spectrophotometrically at 405 nm. The activity was normalised with protein concentration, and then leveled in proportion to the control. For analyzing the putative role of DPP-4, linaglipin was used to inhibit the activity of DPP-4.

### Statistical analysis

The statistical software SPSS v.12.0 was used to analyze the data. One-way ANOVA was performed (p < 0.05), while Bonferroni’s multiple comparison was used for post-test.

## Result

### The applied doses of Aβ and AE subfractions were determined

**Fig 1:**
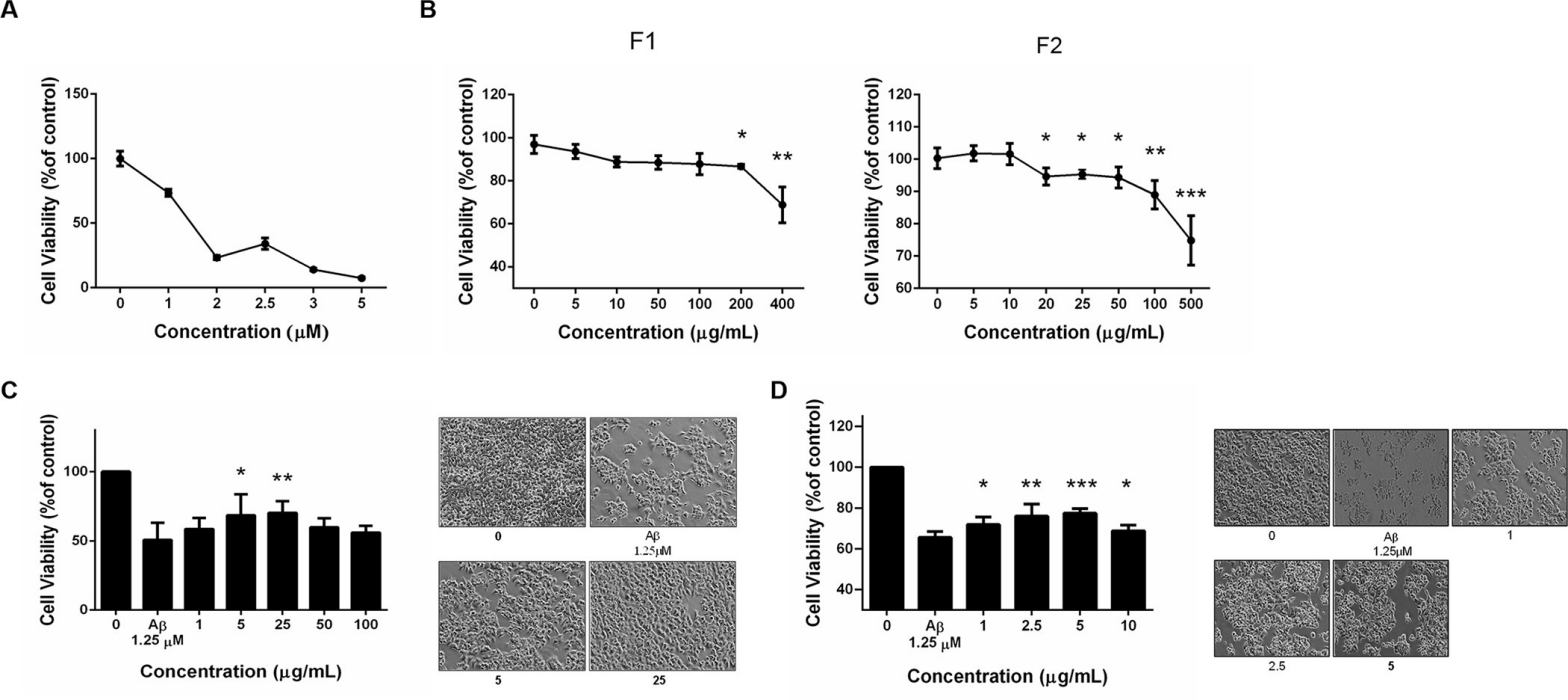
Cytotoxicity test for Aβ and AE subfractions. SK-N-MC cells were incubated for 24 h with or without different concentrations of (A) **Aβ**, (B) F1 and F2. Thereafter 1.25 μM of Aβ was determined to be used in the following experiment, treated with different concentrations of F1 (C) and F2 (D). Cell viability was analyzed with MTT and calculated as a percentage compared with that of the control group. Data were presented as means ± SD (n = 3), and statistically analysed with ANOVA. *p < 0.05, **p < 0.01, ***p < 0.001, compared with the control. Photos revealed the observation under phase contrast microscopy.

At the beginning of the study, we used MTT to analyze the suitable dose Aβ which distinctly influence the cell proliferation but not exhibit a detrimental effect on the cells. Fig 1A showed that above the dose of 1.0 μM and 1.5 μM, Aβ led to more than 30% and 50% of the cell death, respectively. Considering the number of cells remained, and the amount of proteins extractable, the applied dose of Aβ was determined to be 1.25 μM for the following experiment. The MTT was also used to analyze the applicable dose of F1 and F2. It showed that the treatment of F1 and F2 alone should not exceed 100 μg/mL and 10 μg/mL, respectively, or the cell viability could be influenced (Fig 1B).

### AE subfractions inhibited Aβ-induced cell death

The quantification and microscope observation revealed that F1 especially at the dose of 5 μg/mL and 25 μg/mL, did rescue the Aβ-induced cell death, while F1 over 50 μg/mL had no effect (Fig 1C). Similarly, F2 at 1, 2.5 and 5 μg/mL, rescued the Aβ-induced damage dose-dependently. However, the effect became weaker at higher dose (Fig 1D).

### AE subfractions inhibited Aβ-induced apoptosis

**Fig 2:**
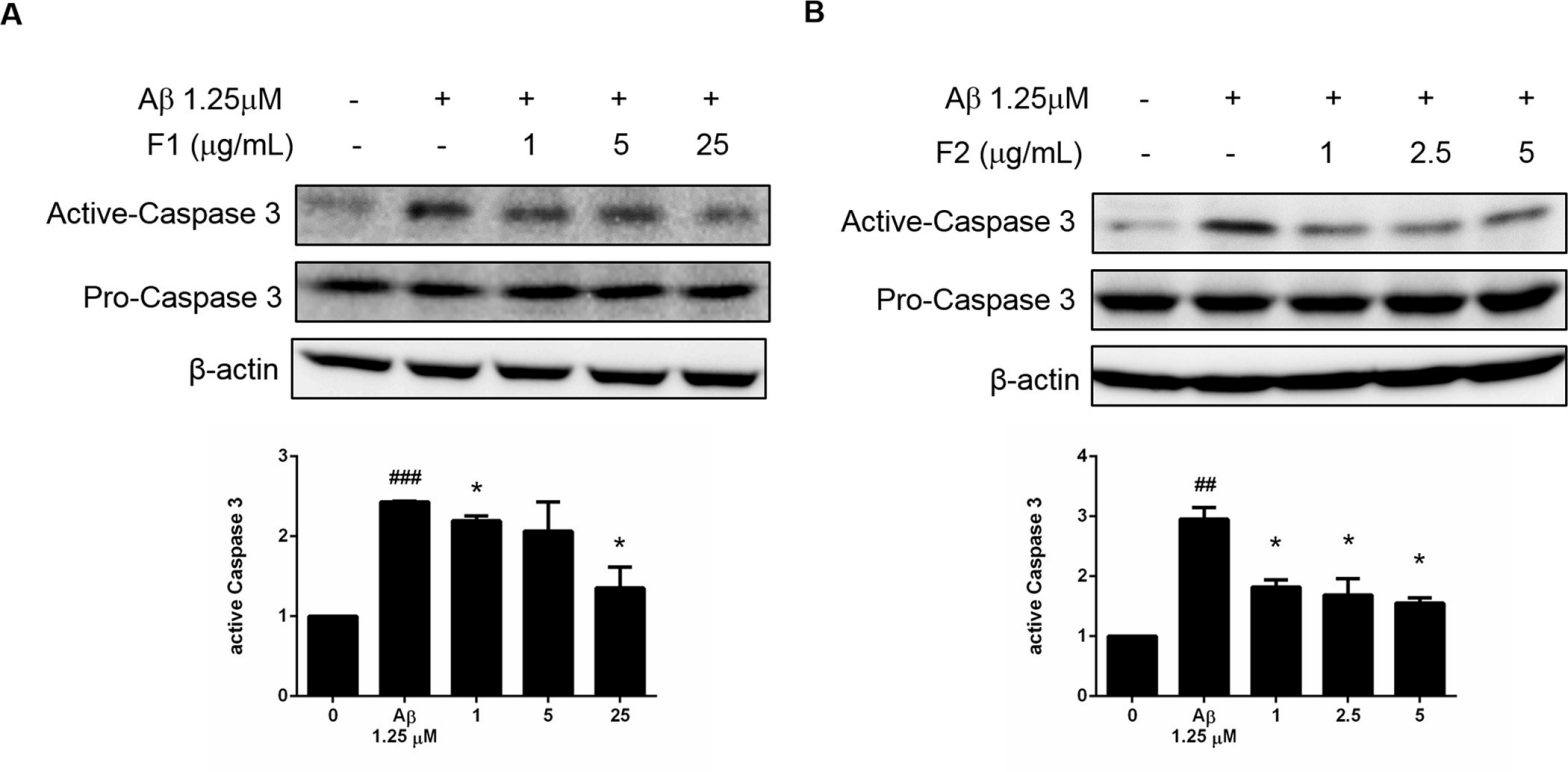
Effect of AE on Aβ-induced apoptosis. SK-N-MC cells were incubated for 24 h with or without Aβ and different concentrations of F1 (A) and F2 (B). The expressions of caspase 3 were analyzed by Western blotting. Data were presented as means ± SD (n = 3), and analysed with ANOVA. #p < 0.05, #p < 0.01, #p < 0.001, compared with the control. *p < 0.05, **p < 0.01, ***p < 0.001, compared with the Aβ-treated only.

The expression of caspase 3 was viewed as common point of different apoptotic pathway. It was demonstrated that Aβ increased about 2.5 folds of the activation of caspase 3. Treatment of F1 dose-dependently decreased the level of cleaved-caspase 3, especially at the dose of 25 μg/mL (Fig 2A). As well, F2 significantly reduced the activation of caspase 3. Evan at the low dose of 1 μg/mL, F2 reduced 40% of the cleaved-caspase 3 compared with the group of Aβ (Fig 2B).

### AE subfractions inhibited Aβ-induced expression of DPP-4, while stimulated the phosphorylation of AMPK and PI3K

**Fig 3:**
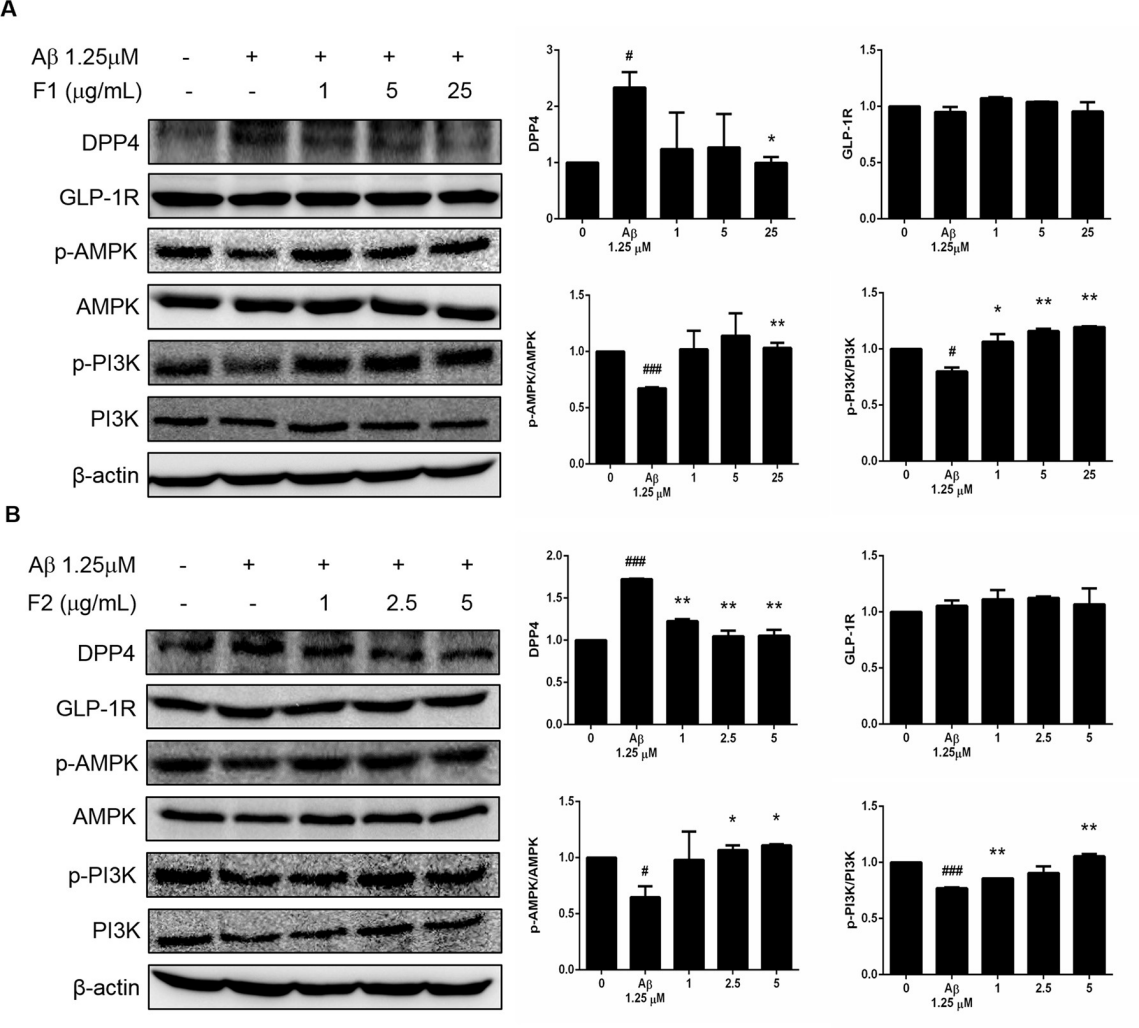
Effect of AE on Aβ-increased DPP-4 and Aβ-decreased phosphorylation of PI3K and AMPK. SK-N-MC cells were incubated for 24 h with or without Aβ and different concentrations of F1 (A) and F2 (B), and then analyzed with Western blot. The protein levels of GLP-1R, DPP-4, p-PI3K and p-AMPK were calculated as a percentage compared with that of the control group. Data were presented as means ± SD (n = 3), and analysed with ANOVA. #p < 0.05, #p < 0.01, #p < 0.001, compared with the control. *p < 0.05, **p < 0.01, ***p < 0.001, compared with the Aβ-treated only.

Fig 3 showed that the treatment of Aβ presented approximately two-fold increase expression of DPP-4 as compared to the control. F1 at 25 μg/mL significantly inhibited DPP-4 to the level of the control. In the contrast, Aβ decreased the phosphorylation of AMPK (p-AMPK) and PI3K (p- PI3K). Treatment of F1 reversed the level of p-AMPK and p- PI3K (Fig 3A). Similarly, Fig 3B also showed that treatment of F2 alleviated the Aβ-increased DPP-4, and reversed p-AMPK and p- PI3Kespecially at the dose of 5 μg/mL. As for GLP-1R, it was not altered either by Aβ or AE subfractions in the present experiment.

### AE subfractions inhibited Aβ-induced phosphorylation of IRS-1-Ser307 and Tau, but stimulated the phosphorylation of GSK-3β

**Fig 4:**
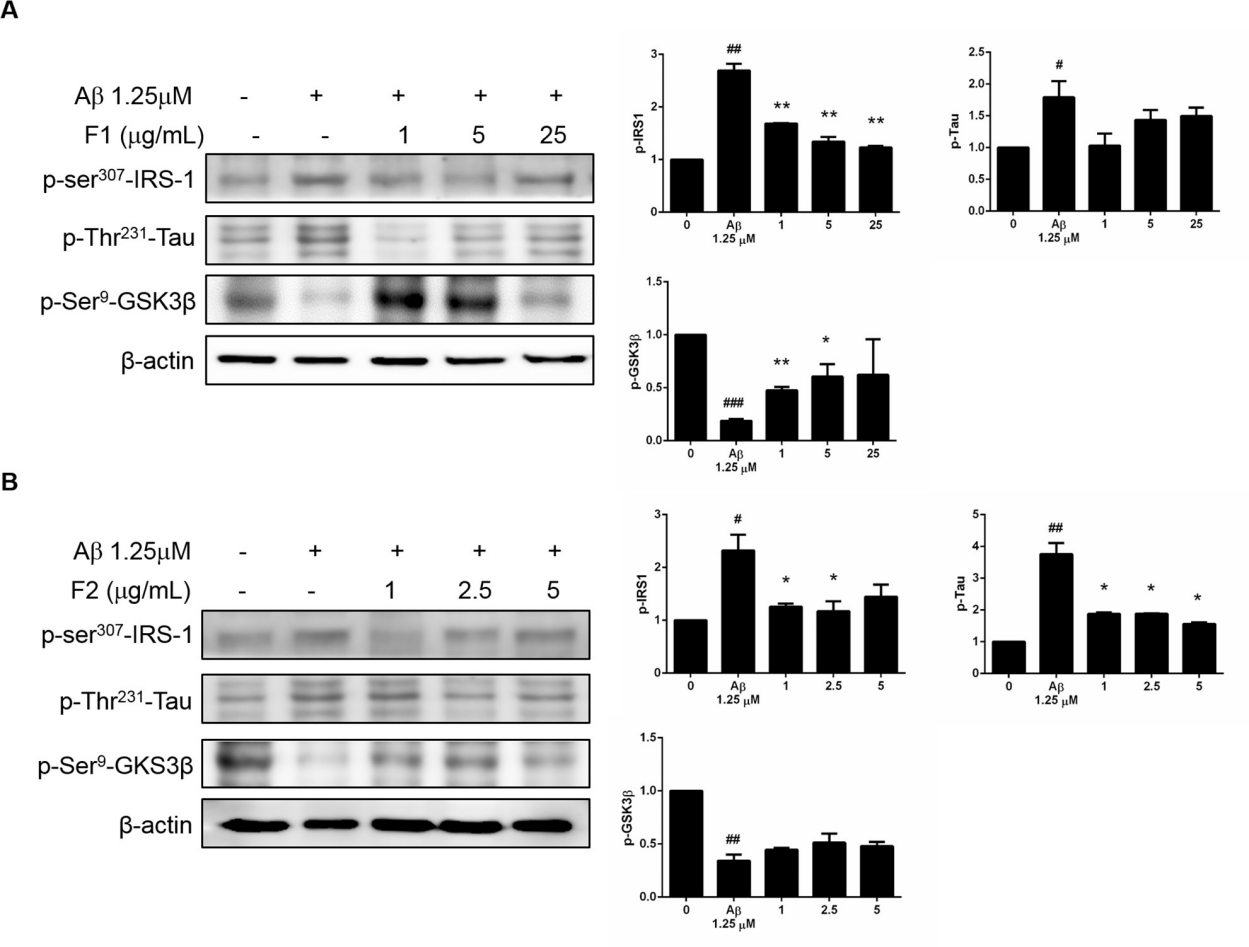
Effect of AE on Aβ-increased phosphorylation of IRS-1-Ser307 and Tau, and Aβ-decreased phosphorylation of GSK-3β. SK-N-MC cells were incubated for 24 h with or without Aβ and different concentrations of F1 (A) and F2 (B), and then analyzed with Western blot. The protein levels of p-IRS-1-Ser307, p-Tau and p-GSK-3β were calculated as a percentage compared with that of the control group. Data were presented as means ± SD (n = 3), and analysed with ANOVA. #p < 0.05, #p < 0.01, #p < 0.001, compared with the control. *p < 0.05, **p < 0.01, ***p < 0.001, compared with the Aβ-treated only.

As shown in Fig 4, Aβ increased the phosphorylation of IRS-1-Ser307. Treatment of F1 and F2 significantly reduced the expressions of p-IRS-1-Ser307. Although both F1 and F2 showed effective to regulate Tau and GSK-3β, F2 still took advantage to reduce the phosphorylation of Tau, while F1 was superior to enhance the phosphorylation of GSK-3β.

### AE subfractions attenuated Aβ-induced activation of DPP-4

**Fig 5:**
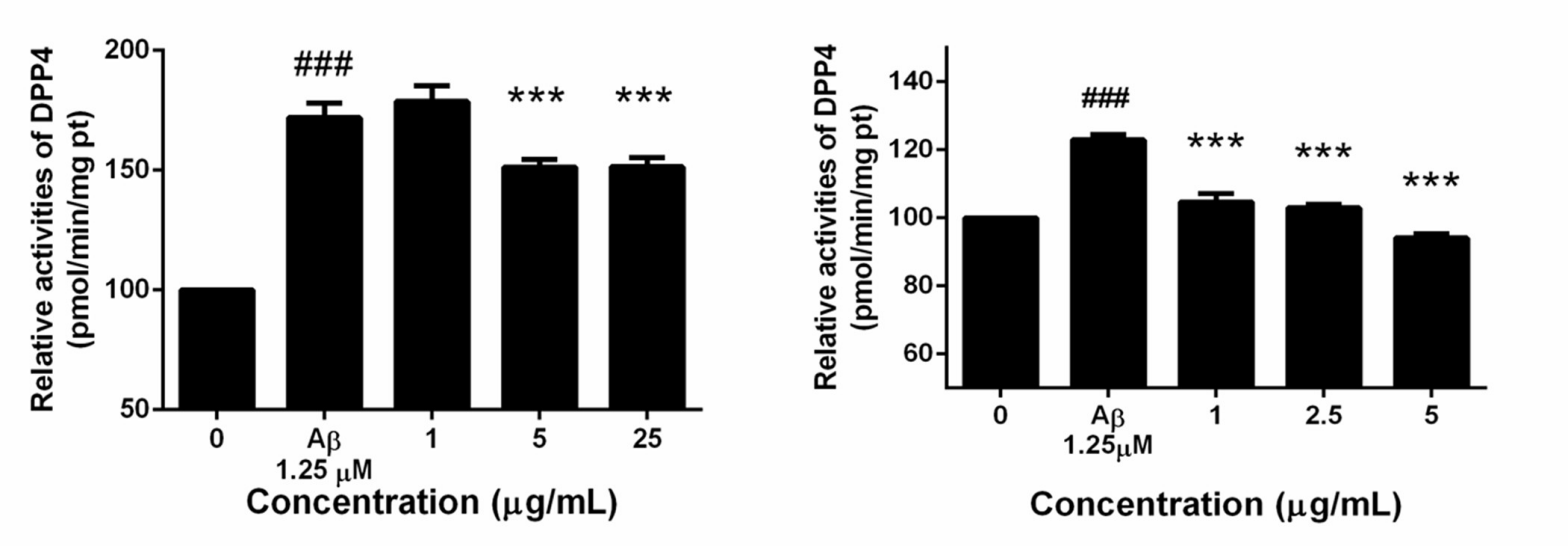
Effect of AE on Aβ-enhanced DPP-4 activity. SK-N-MC cells were incubated for 24 h with or without Aβ and different concentrations of F1 (A) and F2 (B). DPP-4 activity were analyzed and calculated as a percentage compared with that of the control group. Data were presented as means ± SD (n = 3), and analysed with ANOVA. #p < 0.05, #p < 0.01, #p < 0.001, compared with the control. *p < 0.05, **p < 0.01, ***p < 0.001, compared with the Aβ-treated only.

The activity analysis revealed that Aβ, not only increasing the protein level, but did enhance the activity of DPP-4 (Fig 5). Although 1 μg/mL of F1 had no effect, treatment of 5 μg/mL and 25 μg/mL significantly reduce the DPP-4 activity. However, F2 seemed to be more potential. F2 effectively reduce the DPP-4 activity at the low dose of 1 μg/mL. At 5 μg/mL, F2 reduce the DPP-4 activity even below the control.

### DPP-4 plays a critical role in Aβ-induced apoptosis

**Fig 6:**
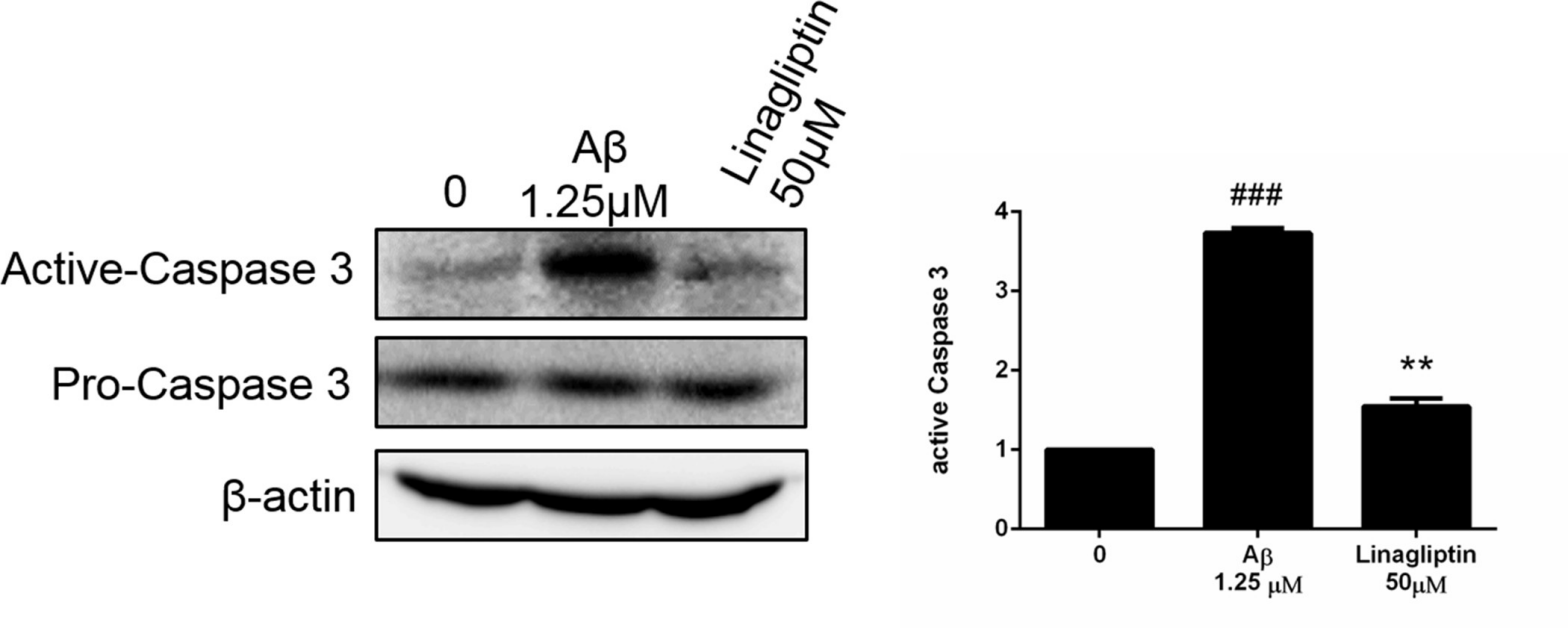
Role of DPP-4 in Aβ-induced apoptosis. SK-N-MC cells were incubated for 24 h with or without Aβ and the DPP-4 inhibitor linagliptin. The protein levels of procaspase and active caspase were analyzed with Western blot and calculated as a percentage compared with that of the control group. Data are presented as means ± SD (n = 3) and analysed with ANOVA. ###p < 0.001, compared with the control. *p < 0.05, **p < 0.01, ***p < 0.001, compared with the Aβ-treated only.

For investigating the putative role of DPP-4, linagliptin was used as the DPP-4 inhibitor (Fig 6). MTT analysis revealed that treatment of linagliptin did not alter the cell viability until 50 μM, which inhibit the activation of DPP-4 more than 20% [S2 File]. Treatment of linagliptin substantially inhibited the Aβ-induced activation of caspase 3, suggesting that DPP-4 should play an important role to mediate Aβ-induced apoptosis of neurons.

## Discussion

**Fig 7:**
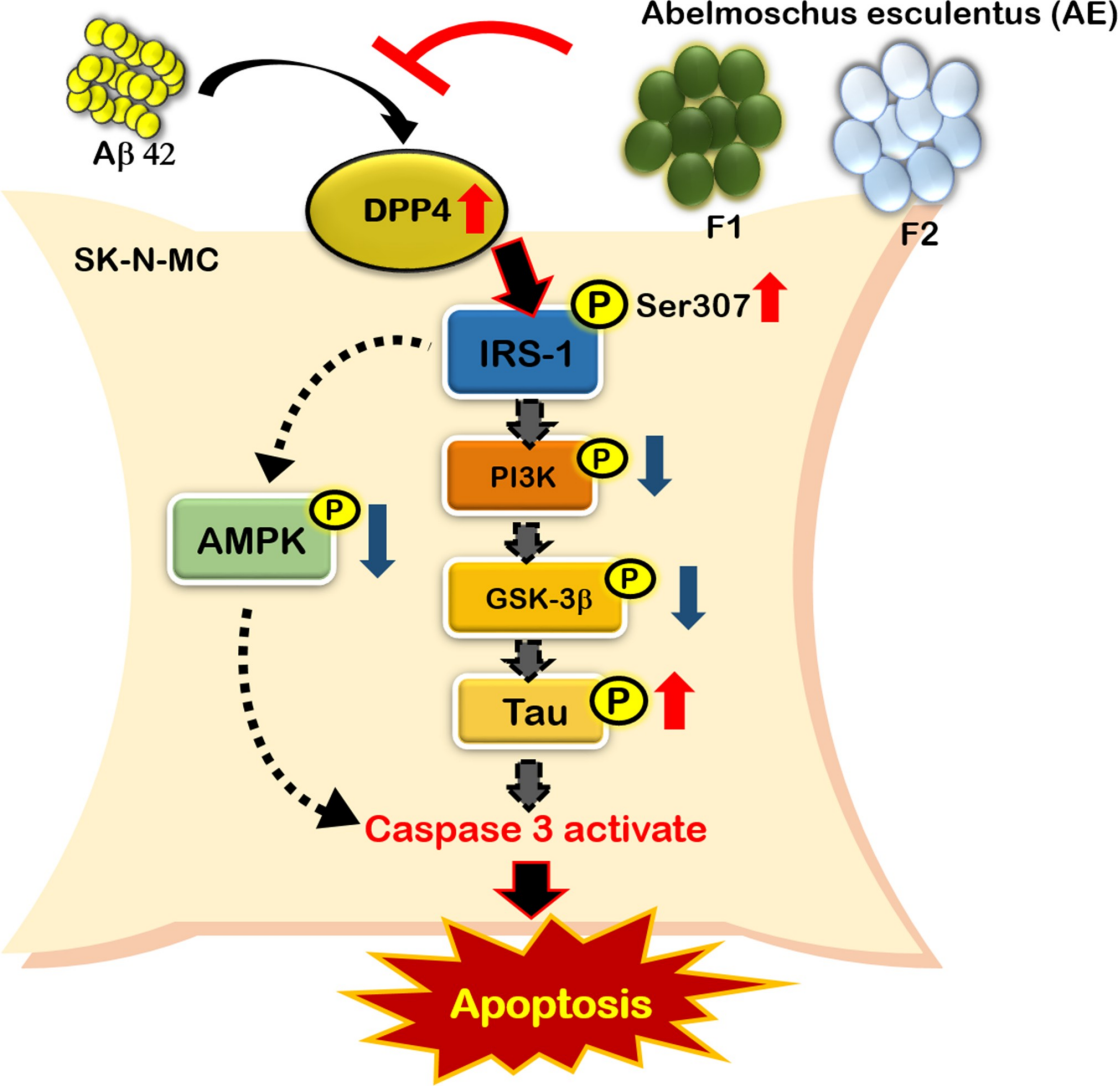
Summary diagram.

In the present study, we demonstrated that the treatment of appropriate dose of AE subfractions inhibit Aβ-induced apoptosis of neurons (Fig 7). Treatment of F1 attenuates Aβ-induced cleavage of caspase 3, while F2 attenuates the activation of caspase 3 even at the low dose of 1 μg/mL. Both AE subfractions decrease Aβ-enhanced expression of DPP-4, but increase Aβ-reduced expression of p-AMPK and p-PI3K. However, although both F1 and F2 inhibit the expressions of p-IRS-1-Ser307, F2 takes advantage to reduce p-Tau, and F1 is superior to enhance p-GSK-3β. In comparison of the AE subfractions, F2 is more potential to reduce the activation of DPP-4, which plays an important role in the Aβ-induced apoptosis.

It is noteworthy that AE subfractions have quite available dose range, while Aβ brings severe toxicity even at tiny quantity (Fig 1A and 1B). F1 and F2 alone could be applied to neurons up to 100 μg/mL and 10 μg/mL, respectively. However, co-treated with Aβ, the valid dose of F1 and F2 against Aβ-induced cytotoxicity would not be higher than 25 and 5μg/mL, respectively (Fig 1C and 1D). It might be speculated the peptide Aβ binds to cell membrane, changes the protein conformation, and thus results in the sensitivity alteration to AE subfractions. Moreover, the response to particular chemical would up to the dose apply. It was reported quercetin glycosides, which is also abundant in our F1, exhibit the antioxidation or ctotoxicity at low or high IC50, respectively [19]. Hence the dose of AE was determined and treated appropriately to attenuate the apoptosis marker in the present *in vitro* model (Fig 2).

Recently, it was reported an oral hypoglycemic agent, vildagliptin, inhibits the expression of DPP-4 and prevents the apoptosis of hippocampal neurons with attenuating the levels of caspase-3, Bcl-2 and associated X protein. Vildagliptin reversed diabetes-induced decrease of p-Akt, p-GSK-3β, brain-derived neurotrophic factor and nerve growth factor, thus against cognitive deficits and memory impairment [20]. It was suggested chronic endoplasmic reticulum (ER) stress can trigger the cell apoptosis. Treatment with vildagliptin promoted β cell survival in db/db mice, with down-regulation of the markers of ER stress including CHOP, TRIB3 and ATF-4 [21]. Another DPP-4 inhibitor gemigliptin has been shown to effectively inhibit ER stress-induced apoptosis in cardiomyocytes, via Akt/PERK/CHOP and IRE1alpha/JNKp38 pathways [22]. In addition, sitagliptin has been shown to attenuate hypoxia-induced apoptosis and autophagy of mesenchymal stem cells [23]. Compared with the literature, although the inhibition of DPP-4 would act on extending the half-life of GLP-1 in vivo, our in vitro findings indicated that DPP-4 per se mediated Aβ-induced apoptotic signal cascades of the neurons (Fig 3, Fig 5 and Fig 6).

Our previous report showed that *Hibiscus sabdariffa* polyphenols (HPE) improved insulin sensitivity by attenuating DPP-4 and the downstream signals in the palmitate-induced model displaying insulin resistance. By decreasing the pIRS-S307 while increasing pPI3K, HPE improved the DPP-4-mediated EMT in renal tubular cells [11]. Recently, we demonstrated that AE decreased apoptosis of β cells. The subfractions of F1 and F2, especially F2, reversed the palmitate-increased DPP-4, which was shown to be critically involved in the apoptotic signaling. AE regulated the cascades of AMPK/mTOR, PI3K and mitochondrial pathways, and prevent the exacerbation of β cells [17]. Hence the regulation of PI3K and AMPK (Fig 3) are speculated to be downstream of DPP-4/p-IRS307, thus mediating the apoptotic cascades.

The activation of PI3K converts membrane phosphatidylinositol-bisphosphate to phosphatidylinositol-trisphosphate, thus activates Akt, leading to the phosphorylation of biologically important substrates including GSK3β. The phosphorylation inactivates GSK3β, which normally promotes cell proliferation and cell survival [8]. GSK3β showed to regulate tau phosphorylation, thereby contributing to the formation of neurofibrillary tangles [24]. Our finding about PI3K, GSK3β and tau (Fig 3 and Fig 4), is consistent with the literature. AMPK is an energy sensor that regulates cellular metabolism. It was reported in aged mice, chronic adiponectin deficiency which is associated with insulin resistance, led to cognitive impairments and Alzheimer's disease-like pathologies through the cerebral AMPK inactivation [25].

The F2 subfraction of AE contains large amount of polysaccharides from the analysis of phenol-sulfuric acid method. Monosaccharide composition analysis indicated that F2 consisted of uronic acid, galactose, glucose and myo-inositol, as well as rhamnose, fucose, and glucosamine [16]. In neuroblastoma cells, the myoinositol phosphate showed to inhibit beta-secretase, which produce Aβ from APP through the sequential cleavage with gamma-secretase [26]. It was reported fucose-rich oligo- and polysaccharides stimulated cell proliferation and promoted cell survival, thus slowing the aging of skin fibroblasts [27]. In addition, the O-linked N-acetylglucosamine (O-GlcNAc) has been shown to modify the subcellular localization and half-life of proteins, the protein-protein interactions, the enzyme activity, and the DNA binding. Although it is still controversial, O-GlcNAc could play a role in the regulation of cell survival [28]. A lipophilic derivative of glucosamine has been shown to prevent the impact-induced chondrocyte death via putative mechanism reducing mitochondrial depolarization [29].

As for F1, it is composed of at least 10 compounds, including quercetin and quercetin glucosides. In focal cerebral ischemia, quercetin was reported to attenuate cell apoptosis via activating the BDNF-TrkB-PI3K/Akt signaling pathway [30]. In addition, quercetin has been shown to regulate apoptotic genes via blocking JNK- and p38 MAPK-related signaling triggered by oxidation, thus prevented apoptosis and promoted cell survival [31]. Quercetin 3-O-methyl ether has been shown to protect liver cells from Cu (2+)-induced apoptosis and mitochondrial dysfunction. PI3K, Akt and Erk were critically involved in the survival of quercetin ether-treated cells [32]. As for the present data, the regulation of PI3K by F1 should be at least partly attributed to the quercetin derivatives.

Our recent report demonstrated that F2 was most effective in attenuating hyperglycemia, insulin resistance, and especially the level of HbA1C, using a type 2 diabetic model. While F2 was associated with weight gain, F1 possessed an anti-obese effect, and the residue of AE had benefit to improve lipoprotein profiles [33]. Furthermore, despite all the AE subfractions ameliorated albuminuria and renal hyperfiltration accompanied with diabetes, F2 acted most promptly and consistently, and reduced oxidative stress on kidneys specifically. These results suggested that AE subfractions could be developed individually and deserve further investigation [34].

In conclusion, we demonstrated AE is potential to prevent Aβ-induced neuron damage by regulating DPP-4 and the insulin resistance cascades. AE, especially F2, deserves further investigation and could be developed as an adjuvant to protect neuron degenerative disease related to Aβ and insulin resistance.

## Supporting information

S1 File Procedures of AE extraction. (TIF)

S2 File Dose and effect of linagliptin. (TIF)
