# Supplementary figures and images for: Abelmoschus esculentus subfractions attenuate beta amyloid-induced neuron apoptosis by regulating DPP-4 with improving insulin resistance signals
Source: PLoS One. 2019 Jun 25;14(6):e0217400. doi: 10.1371/journal.pone.0217400 (PMC6592593; doi:10.1371/journal.pone.0217400)

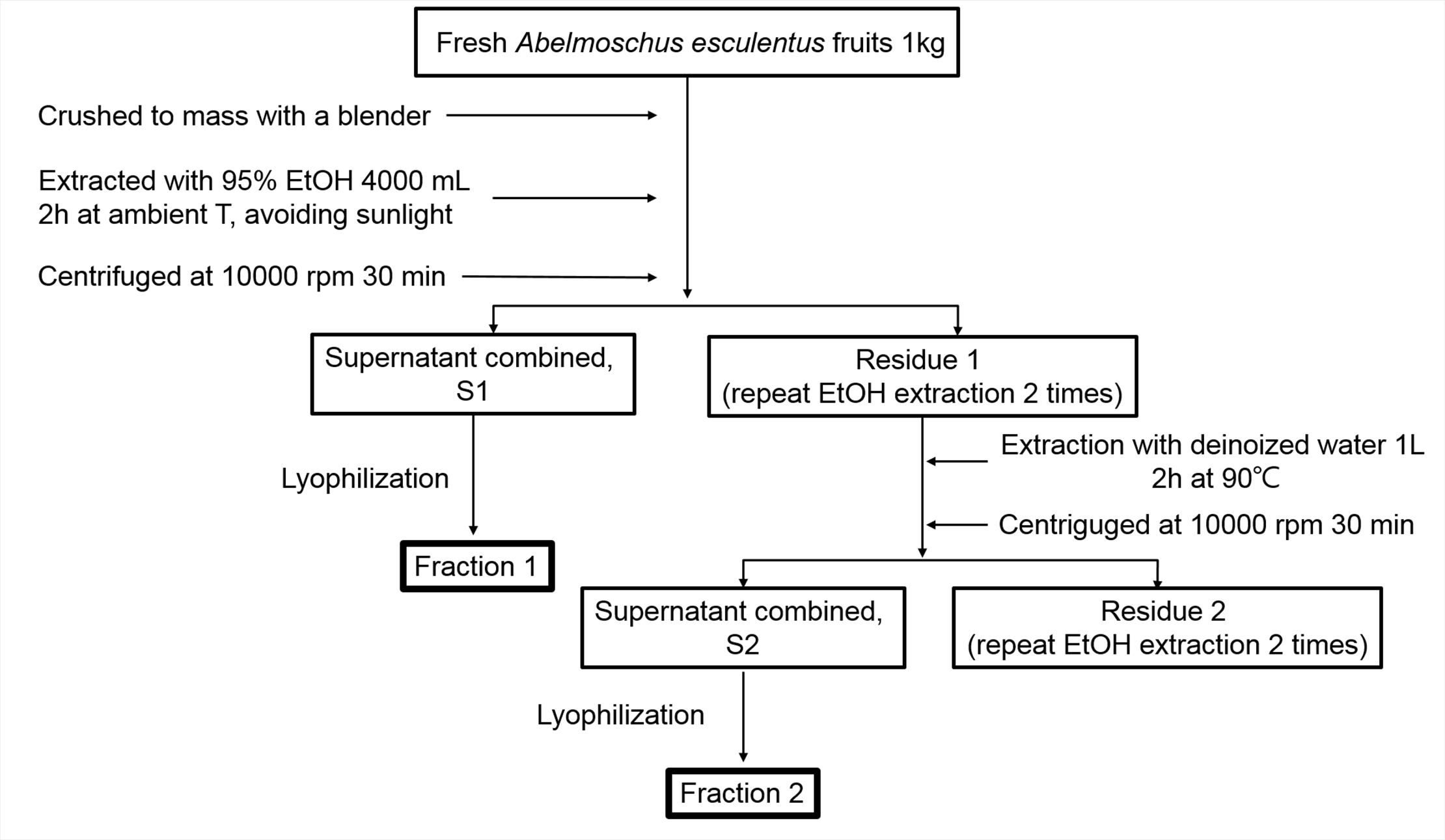

Supplement: S1 File — (TIF) [file pone.0217400.s001.tif]

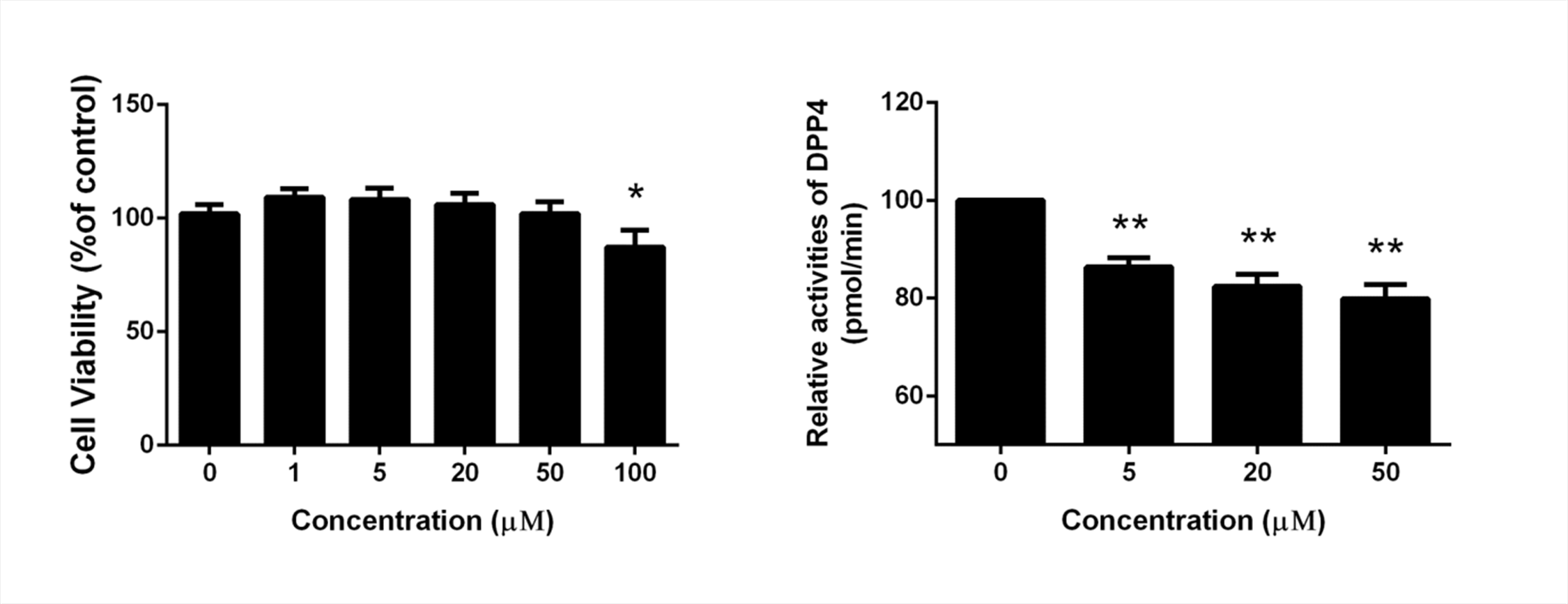

Supplement: S2 File — (TIF) [file pone.0217400.s002.tif]
